# Teething

**Published:** 1872-10

**Authors:** S. J. Cobb


### ARTICLE III.

### *Teething.*

BY S. J. COBB, D. D. S.

As a general thing, our profession is not called upon or expected to do much, until the teeth are pretty well developed; still it may not be amiss for us to make a few suggestions in regard to the eruption of these organs. In looking up statistics upon this subject, we find that fully one-fourth of the human family die cutting teeth. If these statistics are correct, which we do not doubt, it shows teething to be a very important subject, one that should interest everybody.

Cutting teeth is one of nature's own arrangements, that ought to be done with little or no trouble; and would be, but for the violation of some of her laws, the penalty of which must be suffered, whether it cost the life of one-fourth or all the children born.

The question is, who is to be held responsible for the violation of these natural laws? Certainly not the little ones who die so early in life; then it must be the parents, grand-parents, great grand-parents, and parties back as far as the iniquity of one is visited unto another.

We are satisfied that the loss of many of these little children from teething, is the result of the life and habits of their parents; children born of parents whose teeth are sound, and their health perfect otherwise, seldom die from teething. The laws of health should be understood and obeyed. Dr. Guernsey, an excellant medical writer, says: "A large class of diseases arise from ignorance of the laws of health, of the delicate and beautiful organization of the system, of the great end and aim of existence, of the harmonious play, which should ever exist between all the organs and faculties of our being, between the mind and the body, the spiritual and the physical."

Amid the wild and restless scenes of life, the ceaseless whirl and mad excitement of business, the feverish panting for fame, wealth or power; as well as in the more quiet paths of rural life, or the slothful walks of luxurious ease; but very few maintain that beautiful harmony between all the organs, that equilibrum throughout the system which is essential not only to health, but true greatness.

As well might we expect ripe fruit amid the cutting winds, the ice and snow of our northern winter, or the rich luxuriance of southern vegetation in our colder northern clime, as to expect health, a happy temperament, and above all, a clear and strong mind, unless an equilibrum be kept up in the system, and each organ receives its necessary and only necessary degree of attention and cultivation. All this goes to show the importance of early instruction in regard to the laws of health. Among the first lessons learned in life, should be health lessons. We have always thought that health lessons should be taught in schools to such an extent, that no one could pass through the primary department without being pretty well posted in regard to the laws of health.

Rear up and educate in this way for a few generations, and we will not only save the lives of many children that die teething, but much of their trouble from sickness and disease in after life. "As the twig is bent so the tree is inclined." Why not lead our little human twigs healthward so that their minds may be fully developed in that direction? To do so will require a radical change in some of our habits, customs, fashion and mode of living.

Prof. Cutler, a gentleman of fine observation, says, in a paper read before the California State Dental Association: "In large towns and cities we find very little natural life, all being artificial that can be made so; night substituted for day, the very time that nature requires rest and repose, for the purpose of nutrition and normal repair of wasted tissues. Very little physical exercise is taken, the most important hygiene for bone and muscle development, as well as all other anatomical and physiological perfectibility. Unless muscular exercise is fully taken in some form or other, the bones cannot be well developed; they will lack that density and resistance essential to physical endurance.

"The muscles which are more or less attached to the bones, confer great power on them as levers, to bring about the various forms of locomotion, hence the necessity of regular and thorough exercise. In fashionable society there are no boys nor girls; they are all masters and misses; young gentlemen and ladies, making and receiving calls with as much formal state and stiffness as though they were so many machines wound up for the occasion, going through certain prescribed conventional formalities; no juvenile exuberance, hilarity of spirits and flow of youthful impulses. All such would be considered decidedly vulgar and boorish by these young dignitaries."

Those who violate the laws of nature in this or any other way, better change and get back to nature's plain, simple ways. Nature's arrangements are all simple when understood. That accounts for the simplicity of the truly wise, who are always the plainest and simplest of the simple. No better proof of the simplicity of nature's ways need be offered than the almost perfect health of the lower animals, which is attributed to their simple instinctive ways of living. We, in our boasted wisdom and superiority, are the greatest sufferers.

While it is the duty of all to understand and obey the laws of health, it is especially necessary for those who are weakly and predisposed to disease, to observe and practice all the hygienical arrangements that will contribute or promote their health in any way. Delicate, feeble women should pay special attention to their health during the period of child bearing and nursing. They should keep up their fresh air exercise, and pay strict attention to bathing and clothing; be sure to have their clothing easy and comfortable. As to diet, they should live on plain wholesome food. If their teeth should be carious or unsound, they should use unbolted or brown bread; besides they should eat the most of fruit without peeling; in this way they will get plenty of bone material for their children's teeth. And as soon as these children get old enough, say one month old, they should commence their fresh air exercises, and as they advance in age they should be kept in the open air as much as possible, always dressed comfortable. So much of this dress parading of children is injurious to their health, to say nothing of molding and shaping their minds for similar parade in after life. When they get large enough to romp and play in the dirt, allow them the privilege of giving vent to their natural instincts. It is about as natural for children to wallow and play in the dirt, as it is for a pig to wallow in the mud. While cleanliness is of great importnance, it must be remembered that too much of a good thing never fails to result in evil; much of our sickness is the result of the extravagant use of good things. If every body would recognize the fruits as they are, that nature's laws are not only full and complete, but unchangeable, and that each and every one has its penalty, that must be endured when violated, we would have less sickness and disease, which is nothing more nor less than the penalty of violated law. We will mention four great essentials to life and health, that must be taken properly, otherwise perfect health cannot be enjoyed. Fresh air, wholesome food, exercise and sleep, when properly taken, with good regular habits, never fail to give health.

Nothing short of a thorough knowledge and strict obedience to the laws of health will ever save the human family from the vast amount of suffering from sickness and disease. This we offer as the only remedy to save the great number of children that die teething, as well as an equal number that die of other diseases before five years old. The medical profession in its various departments have undoubtedly power to be the greatest benefactors known to man, yet with all its science, skill and ability, it has failed to take half the children born to middle age, and we dare say will continue to fail until children are early instructed into the laws of health. In addition to such lessons as may be taught in school books, which should be extensive, but simple, we would advise every family not only to take but read and make every child read regularly some good health Journal.

